# Erectile Dysfunction in the Elderly: An Old Widespread Issue with Novel Treatment Perspectives

**DOI:** 10.1155/2014/878670

**Published:** 2014-03-17

**Authors:** Pietro Gareri, Alberto Castagna, Davide Francomano, Gregorio Cerminara, Pasquale De Fazio

**Affiliations:** ^1^Elderly Health Care, ASP Catanzaro, Via Spasari, 3, 88100 Catanzaro, Italy; ^2^Geriatrician AUSL Modena, 41120 Modena, Italy; ^3^Department of Medical Pathophysiology, “Sapienza” University of Rome, 00198 Rome, Italy; ^4^Department of Science of Health, School of Medicine, University “Magna Græcia” of Catanzaro, 88100 Catanzaro, Italy; ^5^Psychiatry Unit, “Mater Domini” University Hospital, 88100 Catanzaro, Italy

## Abstract

Erectile dysfunction (ED) is one of the most common chronic diseases affecting men and its prevalence increases with aging. It is also the most frequently diagnosed sexual dysfunction in the older male population. A number of different diseases potentially worsening sexual function may occur in elderly people, together with polypharmacy. Related causes of ED are variable and can include arterial, neurogenic, hormonal, cavernosal, iatrogenic, and psychogenic causes. The aim of the present review was to examine the main aspects of erectile dysfunction going through epidemiology and pathophysiology and revise most of ED in elderly disabled men and in those affected with psychiatric disorders. Lastly we tried to focus on the main aspects of nonpharmacological and pharmacological treatments of ED and the recreational use in the elderly. Phosphodiesterase-5 inhibitors (PDE5-I) are commonly used for on-demand or chronic treatment of ED. It is widely known that PDE5-I have lower response rates in older men than in younger patients, but they have the advantages of ease of use and excellent safety profile, also in the elderly. The old and new PDE5-I as well as the alternative treatments for ED are extensively discussed.

## 1. Introduction

Erectile dysfunction (ED) is one of the most common chronic diseases affecting men and its prevalence increases with aging. It is also the most frequently diagnosed sexual dysfunction in the older male population [1]. ED is defined as the inability of a man to attain and maintain an adequate erection for satisfactory sexual intercourse. It has become an issue only in the late years, because before the 20th century individuals often did not live beyond the reproductive years. Furthermore, elderly men are often affected with several diseases, leading to polypharmacy; many drugs potentially worsen sexual function [2]. This also means that a careful assessment of potential drug-drug interactions is requested [2]. Related causes of ED are variable and can include arterial, neurogenic, hormonal, cavernosal, iatrogenic, and psychogenic causes [3]. It is now widely accepted that ED is predominantly due to underlying vascular causes, particularly atherosclerosis [4].

The aim of the present review was to examine the main aspects of erectile dysfunction going through epidemiology and pathophysiology and revise most of ED in elderly disabled men and in those affected with psychiatric disorders. Lastly we tried to focus the main aspects of treatment of ED and the recreational use in the elderly. In fact an active sexual life is perfectly in agreement with the geriatrics motto that doctors should help patients to add the life in their years and not the years in their life. An extensive Medline search was made using the keywords: elderly, comorbidities, erectile dysfunction, epidemiology, pathophysiology, endothelial dysfunction, phosphodiesterase-5 inhibitors, and polypharmacy. Due to the great amount of studies on this field, we chose only the most recent articles.

## 2. Epidemiology

In a large US study, the proportion of sexually active males declined from 83.7% in the age group 57–64 years to 38.5% in the age group 75–85 years [5]. All epidemiologic studies clearly show an increasing age-related prevalence and severity of ED. Data from the Massachusetts Male Aging Study documented a tripling of the overall probability of complete ED from 5% in men aged 40 years to 15% in men aged 70 years [6]. In the European Male Aging Study (EMAS), performed in eight European centers for the investigation of ED in men aged 40–79 years old, the prevalence of ED was higher in the old age groups, peaking in men 70 years old and older [7]. Some studies have pointed out that normal erectile function is not a prerequisite to remain sexually active [7–9]. Notwithstanding sexual problems are frequent among older adults, they are infrequently discussed with physicians [9]. Asking about sexual health remains difficult or embarrassing for many primary care physicians and at the same time many patients find that raising sexual issues with their doctor is difficult.

However, after the age of 60 years, the ED rate increases independently of comorbidities such as coronary artery disease, diabetes, and hypertension [10]. Furthermore, elderly men are often affected with several diseases and take a lot of drugs, many of which are potentially worsening sexual function. On the other hand, preserving a good sexuality in both old men and women is remarkable for trying to improve their quality of life.

ED is frequently found in the elderly because it is associated with the same underlying risk factors as vascular disease and includes hypertension, diabetes mellitus (DM), hyperlipidemia, smoking, and obesity which are common during aging. Some evidence shows that ED can be greatly improved not only by some drugs such as phosphodiesterase-inhibitors (PDE5-I), but also by treating the risk factors directly [11]. These include cessation of smoking, correction of hyperlipidemia, and amelioration of obesity through weight loss. In fact, all of them result in amelioration of endothelial health [11]. There is a close relationship between ED, aging, and endothelial dysfunction (EDys). Minor risk factors such as inflammation, hypoxia, oxidative stress, and hyperhomocysteinemia are also related to ED and EDys. ED problems due to organic causes comprise up to 80% of cases, while vascular disease is the most common pathophysiology of ED [12]. Data suggest that ED may be an early manifestation of endothelial dysfunction (EDys) in the presence or absence of cardiovascular risk factors (CRF) [13]. Therefore, men with ED may be at increased risk for cardiovascular adverse events and ED may be considered as a sentinel symptom in patients with occult cardiovascular disease (CVD) [14].

## 3. Pathophysiology

ED in aging males is the result of various factors which exert negative effects on multiple levels in erectile biology [15].

First, in the aging male, the vascular supply to the penis is compromised. In humans, postmortem studies have revealed that aging is often associated with increasing degrees of atherosclerotic vascular alterations in the arterial bed of the penis [16].

Second, the relative proportion of *α*_1_-adrenergic receptor subtypes is modulated by aging in arteries. This means that a lot of age-related changes are found in the human prostatic, bladder, and erectile tissue [17]. Importantly, phenylephrine appears to be less effective in inducing contractions of vascular smooth muscle strips* in vitro* and this is significantly greater for those isolated from the corpus cavernosum of older (>60 years) men with ED than for those isolated from younger (<60 years) men with ED [18]. Another remarkable factor closely contributing to impaired vasodilation in the corpus cavernosum and the penile arterial supply of the older man is endothelial dysfunction. In fact, erectile function is dependent on nitric oxide (NO) production by penile endothelium and thus ED is associated with reduced plasma NO levels [11]. Deficiency of endothelial-derived NO is also believed to be the primary defect that links insulin resistance and EDys [11]. Clinical and biochemical markers of EDys include (1) reduced expression and activity of endothelial nitric oxide synthase (eNOS), reduced synthesis of NO, and increased production of the asymmetric dimethylarginine (ADMA), a competitive, endogenous inhibitor of eNOS; (2) increased production of free radicals of inflammatory cytokines such as interleukin-6 (IL-6), C-reactive protein (CRP), and tumor necrosis factor-*α* (TNF-*α*) and increased endothelial apoptosis [11, 19]. In fact, endothelial dysfunction in aging has been attributed to the presence of NO scavengers in the corpus cavernosum, the most obvious candidates being superoxide anions, whose production is augmented in aging endothelial cells [20]. This causative link is further strengthened by the fact that use of inhibitors of cyclooxygenase and vitamin C, which are both powerful antioxidants, can prevent age-related endothelium-dependent vasodilation decrease in humans [20].

Furthermore, the presence of reactive oxygen species (ROS) is able to cause an inflammatory state of the endothelium resulting in predisposition to atherosclerosis, thereby further reducing blood flow to the erectile tissue. In the aged endothelium, this further results in the inactivation of endothelium NO synthase (eNOS) through a decrease in phosphorylation of its positive regulatory site and an increase in phosphorylation of its negative regulatory site [21]. Animal models represented by aged rats have clearly shown that a decreased activity of eNOs is also responsible for the increase in apoptosis of the endothelium. Therefore, in summary, dysfunctional penile endothelium = reduction in NO release = increased vascular and sinusoidal smooth muscle tone [15].

Third, the percentage of smooth muscle steadily decreases with aging [11, 22]. In fact, corpora cavernosa of aged men present excessive deposition of collagen fibers which results in corporal fibrosis. These changes are similar to those ones observed in the media of the penile arteries [23]. It has been postulated that these histologic changes in the aged corpora, as well as endothelial dysfunction, are caused by increased oxidative stress and/or other profibrotic factors that stimulate smooth muscle apoptosis and collagen deposition [15, 16].

These alterations result in an impaired expandability of the erectile tissue, and therefore the mechanism by which the expanding sinusoids compress the emissary veins against the tunica albuginea becomes defective. This leads to corporeal venous leakage which typically presents as the inability to maintain an erection as it is frequently seen in the aged male.

Finally, another factor contributing to the above described changes in smooth muscle and collagen content of the corpus cavernosum is androgen deficiency. In fact, this can lead to a marked increase in connective tissue deposition. Moreover, venoocclusive dysfunction might be due to an increase in fat containing cells in the subtunical region of penile tissue sections, as shown from orchiectomized animals [24]. Overall, the presence of androgens regulates the normal morphology and function of the cavernous nerves and keeps the endothelium in a healthy condition. A low testosterone (T) level is positively associated with the presence and severity of atherosclerosis and a reduction in plasma T might contribute to increased arterial stiffness, which in turn has been associated with increased cardiovascular risk and mortality [25]. The Rotterdam study, a population-based cohort study, showed that low levels of endogenous androgens are associated with increased likelihood of atherosclerosis in elderly men [26]. Low T was linked to cardiovascular mortality, morbidity in men of varying age, and cardiovascular risk factors (CRF) [27]. Men have a higher rate of CVD than women [11]. The possible culprits appear to be T, dihydrotestosterone (DHT), dehydroepiandrosterone (DHEAS), and their metabolites [11]. However, randomized controlled trials (RCT) have clearly documented that DHEAS is not useful for ED elderly subjects [28]. The role of androgens in determining vasodilation has been recently investigated. T may activate the endothelium and stimulate the NO-cyclic guanosine monophosphate and/or the hyperpolarization-mediated vascular relaxation pathway and may thus add potential beneficial effects on coronary artery atherosclerosis. Additional endothelium-independent effects of T may involve inhibition of the signaling mechanism of vascular smooth muscle contraction, such as intracellular concentration [Ca^2+^] and protein kinase C [11, 29]. However, the final role of T has still to be recognized, since three different meta-analyses have documented an association between low T and CV mortality but not with CV events [30–32]. In particular, low T has been linked to increased blood pressure, dyslipidemia, atherosclerosis, arrhythmias, thrombosis, endothelial dysfunction, and impaired left ventricular function. On the other hand, treatments with T to restore “normal concentrations” have so far neither been proven to be beneficial with respect to cardiovascular disease nor have definitely shown specific adverse cardiovascular effects [30]. Recently, Isidori et al. [33] reported that molecular and clinical evidence supports the use of testosterone replacement therapy (TRT) in hypogonadal patients with ED, although the benefit-risk ratio is uncertain in advanced age. The development of a pathophysiology-oriented algorithm designed to avoid inappropriate treatments and support whether to start with TRT, PDE5-I only, or both is requested, in order to improve diagnosis and individualize a correct management [33]. On the same line, it has been shown that, in late-onset hypogonadism (LOH), TRT is able to improve central obesity in patients affected with metabolic syndrome (MetS) and glycometabolic control in patients with MetS and type-2 diabetes mellitus as well as to increase lean body mass, along with insulin resistance and peripheral oxygenation [34, 35]. Importantly, the increased waist circumference is the major determinant of MetS-associated hypogonadism, whereas androgen deprivation increases abdominal adiposity. Moreover, in cross-sectional studies longitudinal evidence has shown that low T is associated with a higher risk of subsequent development of MetS, although the reverse condition is also possible [34, 35]. In summary, subjects with MetS have lower levels of total T (TT) (about 3 nmol/L), and hypogonadism is more evident in subjects with, than in those without, ED. It has not yet been clarified which are the possible factors in MetS responsible for the low T [34]. However, it should be recognized that the number of studies on benefits of T supplementation is too limited to draw final conclusions [35]. The confusion still continues, due to the recent results of a retrospective national cohort study of men with low testosterone levels (<300 ng/dL) who underwent coronary angiography in the Veterans Affairs system between 2005 and 2011. The aim was to assess the association between testosterone therapy and all-cause mortality, myocardial infarction, or stroke. Of the 8709 men with a total testosterone level lower than 300 ng/dL, 1223 patients started testosterone therapy after a median of 531 days following coronary angiography. The absolute rate of events was 19.9% in the no testosterone therapy group versus 25.7% in the testosterone therapy group, with an absolute risk difference of 5.8% (95% CI, −1.4% to 13.1%) at 3 years after coronary angiography. Therefore, the use of testosterone therapy was associated with increased risk of adverse outcomes. No significant difference in the effect size of testosterone therapy among those with and without coronary artery disease (test for interaction, *P* = 0 .41) was reported [36].

## 4. ED in Elderly Disabled Men 

A number of related heart conditions are common causes of ED, including hypertension, dyslipidemia, atherosclerosis, heart disease and, among metabolic diseases, diabetes. Stroke, Parkinson's disease, and multiple sclerosis are other potential causes of ED. Some types of physical trauma and injuries, especially those affecting the pelvic area or spinal cord, may cause nerve damage leading to erectile dysfunction. Orthopedic surgery, fistula surgery, and surgeries on prostate, colon, or rectal cancer can also significantly contribute to the decrease of sexual function. All of these conditions are very frequent in elderly patients; comorbidities and polytreatment are a hard challenge in elderly people health management. Furthermore, in the elderly, we have to distinguish the effects of a number of different diseases and/or their treatments on erectile function and the effects which the same diseases can have on functional independence. In fact, sexual function requires abilities in movements which can be hampered. In the following section we will try to examine in detail some different diseases potentially able to interfere with sexual function in the elderly.

### 4.1. Diabetes

Data collected from 1,195 randomly selected, community-dwelling men as part of the Florey Adelaide Male Ageing Study showed that with increasing age diabetes appears to be independently associated with moderate-to-severe ED [37]. In particular, several studies have shown that lower levels of glycated hemoglobin and therefore a good glycemic control are able to reduce the prevalence of ED and its severity [38–40]. In any case, phosphodiesterase-5 inhibitors (PDE5-I) improve ED in diabetic men [40, 41].

### 4.2. Hypertension

The prevalence of ED varies between 15 and 25% in people affected with hypertension [42]; hypertension is very common in the elderly and is a well-known risk factor for cardiovascular events. ED is reported more than twice as often in men with systolic blood pressure (SBP) > 140 mmHg than in men with SBP < 140 mmHg. Importantly, pulse pressure, that is, the difference between systolic and diastolic blood pressure, an index of arterial stiffness, was suggested to predict incident major cardiovascular events in patients affected with ED [43, 44]. In a consecutive series of 1,093 (mean age 52.1 ± 13.0 years) male patients with ED and without a previous history of hypertension or not taking any antihypertensive drugs it was shown that elevated PP is associated with arteriogenic ED and male hypogonadism [44]. Furthermore, the prevalence of overt hypogonadism (calculated free testosterone < 180 pmol/Lor free testosterone < 37 pmol/L) increased as a function of PP quartiles (17.1% versus 39.7%, and 30.8% versus 58.6% for the first versus fourth quartile, respectively, for calculated free testosterone and free testosterone; all *P* < 0.0001 for trend) [44].

### 4.3. Cardiovascular Diseases

ED may predict the onset of cardiovascular events from 2 to 5 years earlier; both conditions share the same pathogenetic mechanism, which is endothelial dysfunction [43]. Congestive heart failure (CHF) is another frequent disease in elderly patients potentially leading to ED. A sufficient control of symptoms is able to improve sexual dysfunction; if this approach does not work, PDE5-I are the first-line therapy [45]. They can also indirectly improve depressive symptoms and quality of life and can be used in CHF patients classified as New York Heart Association (NYHA) II and III. Of course, sexual activity is contraindicated in patients classified as NYHA IV.

ED ranges from 42% to 75% in patients affected with coronary artery disease and PDE5-I can also be safe and effective in these men [46].

### 4.4. Spinal Cord Injuries

Spinal cord injuries can be present in elderly men as a result of juvenile or recent trauma; PDE5-I are also safe and effective options for these men.

### 4.5. Stroke

The sexual desire, erectile, and ejaculatory functions are impaired after stroke. A lack of sexual desire is the major cause of an absence of sexual intercourse. The specific locations of the stroke lesions, such as the left basal ganglia and right cerebellum, might be associated with sexual desire and ejaculation disorder, respectively [47]. In a survey on 109 stroke patients (mean age 64.93 ± 8.81 years) the lack of sexual desire was the largest cause (59.4%) of an absence of sexual intercourse [47]. Of course, the complete or partial inabilities in moving are a further obstacle other than sexual dysfunction* per se*, when elderly patients are affected with spinal cord injuries or stroke. This on turn can lead to depression, feelings of inutility, and impairment of the ability to recover.

### 4.6. Multiple Sclerosis

Sexual dysfunction (SD) is also a frequent problem for multiple sclerosis patients and appears to be associated with gender. In fact women report more SD than men. Overall, this is another disease where the emotional dimension of SD is related to disability in the aged men [48].

### 4.7. Parkinson's Disease

SD is common and often underrecognized in patients with Parkinson's disease (PD), playing a major role in the deterioration of quality of life of patients and their partners. Loss of desire and dissatisfaction with their sexual life are encountered in both genders and worsen concomitantly to the progression of Parkinsonian symptoms. Hypersexuality, erectile dysfunction, and problems with ejaculation are found in male patients.

Bladder dysfunction (urinary urgency/frequency), bowel dysfunction (constipation), and sexual dysfunction (erectile dysfunction) (also called “pelvic organ” dysfunctions) are common nonmotor disorders in PD [49]. Hypothalamic dysfunction is mostly responsible for the sexual dysfunction (decrease in libido and erection) in PD, via altered dopamine-oxytocin pathways, which normally promote libido and erection. The pathophysiology of the pelvic organ dysfunction in PD differs from that in multiple system atrophy [49]. A relationship among ED, perception of patients' sexual life, and depression is often found in elderly patients affected with PD [50]. Optimal dopaminergic treatment should facilitate sexual encounters of the couple and appropriate counselling diminishes some of the problems (i.e., reluctance to engage in sex and problems with ejaculation) [51].

### 4.8. Dementia

At present ED is often underrecognized and undertreated in dementia and few data are available. Loss of desire can be present in different forms of dementia, whereas hypersexuality can be found in the early-to-moderate stages of frontal dementia. In particular, hypersexual behavior may be a particular feature of behavioral variant frontotemporal dementia (bvFTD), which affects ventromedial frontal and adjacent anterior temporal regions specialized in interpersonal behavior. A recent study reviewed 47 patients with bvFTD compared to 58 patients with Alzheimer's disease for the presence of heightened sexual activity to the point of distress to caregivers and others. Hypersexual behavior occurred in 6 (13%) bvFTD patients compared to none of the AD patients [52]. One patient, with early and predominant right anterior temporal involvement, was easily aroused by slight stimuli, such as touching her palms [52].

## 5. ED in Elderly Men Affected with Psychiatric Disorders 

ED is often the cause of depressive disorders in elderly people [28]. Andropause, ED, and psychiatric disorders often share specific physical and psychological symptoms which complicate the clinical management of elderly men. In particular, anxiety disorders and depression are more frequently linked to sexual dysfunction.

### 5.1. Mood Disorders

Mood disorders may enhance the risk of ED in elderly people. Depressive symptoms are related to sexual dysfunction more frequently than anxiety symptoms. However, according to some authors, ED in elderly psychiatric patients seems to be the expression of androgenic deficit rather than psychiatric symptoms* per se* [53].

ED, the perception of the quality of patient's sexual intercourse and his subjective satisfaction, becomes worse with increasing depressive symptoms. Moreover, in elderly psychiatric patients, symptoms related to hypogonadism have a relationship with ED and both of them can influence sexual performance [54].

Recently some biopsychosocial risk factors have been considered to be responsible for ED. Erectile function worsens with increasing age and fat abdominal mass. Furthermore, the lack of a regular partner, alcohol abuse, and last but not least the presence of depressive and anxiety symptoms worsens its severity [37]. A sample of 203 subjects aged between 45 and 74 years old was assessed through the administration of International Index of Erectile Function 5 (IIEF-5) and Geriatric Depression Scale (GDS), in order to define the presence of a relationship between depressive symptoms and ED severity. ED was shown to be closely linked to depressive symptoms, together with some factors such as life style, smoking, and alcohol [55].

Hypogonadism, ED, and premature ejaculation show significant correlations with physical and mental health in men and in particular with quality of life, metabolic syndrome, cardiovascular diseases, and depressive symptoms [56].

Definitely concomitant ED and depression are really very high; the temporal relationship between these disorders may also be inverted. In other words, ED may be the cause or the consequence of depressive disorder. Men with severe depression have a twofold probability for ED compared to nondepressed subjects [57]. Depression as the consequence of ED was assessed in a recent Canadian study together with the effect of sildenafil citrate, PDE5-I, in patients with untreated depressive symptoms [58].

On the other hand, pharmacological treatment of depressive disorder, in particular with selective serotonin reuptake inhibitors (SSRI), may represent another cause of ED and negatively influence patient's quality of life, his self-esteem, and the relationship with his partner [59].

### 5.2. Bipolar Disorder

ED in elderly psychiatric patients is not only found in depressive and anxiety disorders, but also can represent the complication of antipsychotic treatment in long lasting treatment of bipolar disorder.

ED was found in 42% out of patients affected with bipolar disorder remitted or clinically stabilized by the use of antipsychotics. First generation of antipsychotics is more frequently involved in sexual performance worsening, compared to second-generation drugs [60].

Lastly, ED may represent a frequent symptom in somatization disorders, especially in men of over 45 years old [61].

## 6. Treatment Strategies in ED and Novel Perspectives

Treatment strategies include nonpharmacological and pharmacological procedures. Nonpharmacological treatment includes counseling, life style changes, and medication changes, because a lot of drugs taken by old people can result in negative interference on sexual function.

### 6.1. Counseling

An open communication with the patient and his partner is the first step to set realistic outcome goals. Patient and his partner need to be educated about the anatomy and physiology of sexual function and the right understanding of the pathophysiology of ED. Current oral pharmacological treatments for ED do not “cure” ED but can generally improve erectile function in patients without important comorbidities or underlying disorders such as diabetes or after radical prostatectomy. Furthermore, due to comorbidities and multiple pathophysiologic changes in the old patient's penis, vasculature, and nervous system, a fully rigid erection will be hard to be accomplished with the use of the oral pharmacotherapy alone [15]. Therefore, it is remarkable to provide full information to the couple about the possible pharmacological and mechanical treatment options. The choice of treatment should be made by the patient and his partner supported by the physician, who ideally does not assume an authoritative role in this decision process [62]. In order to preserve mental health and referral to a sexologist or relationship therapist for more extensive counseling, alternative forms of intimacy that do not rely on penetrative sexual intercourse but are as well satisfactory might be helpful [63]. Furthermore, in some patients with psychogenic or mixed psychogenic-organic ED, specialized psychosexual therapy may help relieve anxiety and remove unrealistic expectations associated with medical or surgical therapy [62].

### 6.2. Life Style Changes

Obesity, sedentary life and smoke are related to a higher incidence of ED. First of all, at any age, patients should be educated about the beneficial effects of weight loss, increasing exercise, and quitting smoking on erectile function. In particular, a meta-analysis of 24 studies showed that weight loss is associated with an increase in both bound and unbound testosterone levels. Overall, both a low-calorie diet and bariatric surgery are associated with a significant (*P* < 0.0001) increase in plasma sex hormone-binding globulin-bound and -unbound testosterone levels (total testosterone, TT), with bariatric surgery being more effective in comparison with the low-calorie diet (TT increase: 8.73 (6.51–10.95) versus 2.87 (1.68–4.07) for bariatric surgery and the low-calorie diet, respectively; both *P* < 0.0001 versus baseline) [64]. The normalization of sex hormones induced by body weight loss might be a mechanism contributing to the beneficial effects of surgery in morbid obesity. Moreover, androgen rise seems to be greater in those patients who lose more weight as well as in younger, nondiabetic subjects with a greater degree of obesity [64].

Another systematic review and meta-analysis of randomized controlled trials with a follow-up of at least six weeks evaluated the effect of lifestyle interventions and pharmacotherapy for cardiovascular risk factors on the severity of ED [65]. Lifestyle modifications and pharmacotherapy for CV risk factors were associated with statistically significant improvement in sexual function (IIEF-5 score).

Furthermore, the addition of a statin to men suffering from ED and hypercholesterolemia has shown beneficial effects on erectile function [66].

### 6.3. Medication Changes

A number of different medications can contribute to the cause or worsening of ED such as diuretics, particularly thiazides, and central antihypertensives such as *α*-adrenergic antagonists and nonspecific *α*-antagonists. Tricyclic antidepressants have been linked to both loss of libido up to 70% and ED in 1.7–6.4% [67, 68]. Furthermore, they increase ejaculatory latency time and are currently even prescribed for the treatment of premature ejaculation.

In a study on 344 patients treated with selective serotonin reuptake inhibitors [68], paroxetine provoked more delay of orgasm or ejaculation and more impotence than fluvoxamine, fluoxetine, and sertraline (chi square, *P* < 0.05). Only 24.5% of the patients had a good tolerance of their sexual dysfunction [68]. Twelve male patients who suffered from premature ejaculation before the treatment reported delayed ejaculation, but their sexual satisfaction clearly improved. A positive correlation was shown with dosage and treatment interruption.

Androgen blockers used for treatment of prostate cancer can worsen erectile function and libido. Antihistamines, nonsteroidal anti-inflammatory drugs, antiarrythmics, and drugs for the treatment of Parkinson's disease have been linked to ED.

Importantly, we underline the role of geriatrician in changing drugs potentially causing sexual dysfunction or at least changing its dosage. Another crucial factor is to know the drug interactions potentially able to increase the effects of drugs influencing erectile function [2]. Geriatrician ought to interrupt patient's nonessential drugs with negative impact on erectile function and possibly replace essential drugs by their counterparts or with drugs from another family with less impact on erectile function.

As a matter of fact, treatment of hypertension with calcium channel blockers and angiotensin converting enzyme inhibitors may reverse ED in some patients; switching to an *α*_1_-specific agonist, such as doxazosin, preserves erectile function [15]. Angiotensin receptor blockers such as candesartan, losartan, and valsartan seem to have beneficial effects on erectile function [15].

Alcohol and other abused drugs such as amphetamines, cocaine, marijuana, and opiates have been linked to ED, and the patient should be counseled about these facts [15, 62]. A list of the drugs potentially causing or worsening sexual function are shown as follows:

- abused drugs (amphetamines, opiates, cocaine, marijuana, nicotine, and heroin);
- alcohol;
- antiarrythmics;
- antidepressants (tricyclics, SSRI, and MAO-inhibitors);
- antihistamines (dimenhydrinate, diphenhydramine, and promethazine);
- antipsychotics: butyrophenones (haloperidol) and phenothiazines (promazine);
- barbiturates;
- benzodiazepines;
- *β*-blockers (dose-dependent; propranolol, atenolol, and carvedilol in decreasing order; nebivolol seems to have beneficial effects);
- central antihypertensives;
- 5*α*-reductase inhibitors;
- digoxin;
- diuretics (thiazide diuretics);
- drugs for Parkinson's disease;
- fibrates (clofibrate, gemfibrozil);
- H_2_-blockers (cimetidine, ranitidine);
- Lithium;
- Muscle relaxers;
- Nonsteroidal anti-inflammatory drugs.

### 6.4. Pharmacological Treatment

Pharmacological treatment of ED includes a number of drugs; phosphodiesterase-5 inhibitors (PDE5-I), yohimbine, an *α*_2_-antagonist, PGE1, and papaverine. Intraurethral PGE1 (MUSE), vacuum constriction device, and surgery (penile prosthesis) are other possible therapeutic opportunities.

#### 6.4.1. Use of Phosphodiesterase-5 Inhibitors (PDE5-I) in Elderly Men

The advent of safe and effective oral treatment of ED by PDE5-I has brought a great attention to the disease. In fact, in the elderly, PDE5-I are commonly used for on-demand or chronic treatment of ED and are one of the first-line treatments for patients complaining of ED [69]. As above mentioned, owing to the great number of drugs often taken by geriatric population, a careful assessment of potential interactions between PDE5-I and other drugs is requested. At present, four PDE5-I (sildenafil, vardenafil, tadalafil, and avanafil) are approved worldwide, and two agents (udenafil and mirodenafil) are approved only in Korea [2].

It is widely known that PDE5-I have lower response rates in older men than in younger patients [70], but they have the advantages of ease of use and excellent safety profile, also in the elderly [15, 71]. They are nonhydrolysable analogs of cGMP acting as intracellular signal amplifiers. They work by slowing the degradation of cGMP by PDE5, leading to subsequent penile smooth muscle relaxation. The endogenous nitric oxide (NO), the release of which is evoked by sexual stimulation and is a neurologically mediated event, is decreased during aging. It is further diminished by comorbidities such as hypogonadism, diabetes, and atherosclerosis [70].

All PDE5-I appear to be roughly equivalent in efficacy, with some substantial differences in absorption after oral administration and duration of effect. Vardenafil orodispersible preparation is reported to have the fastest onset of action, that is, as fast as 15 minutes, while tadalafil is reported to have the longer duration of action, that is, as long as 36 hours (more details are reported below) [2]. Sildenafil and vardenafil (film-coated tablets) have only a limited oral bioactivity (about 40% and 15%, resp.) because of extensive presystemic metabolism in the gut wall and liver via CYP3A4 and/or CYP3A5 pathways [40]. This aspect should be carefully considered when planning an ED treatment because it can influence the window of opportunity available for sexual activity [40]. Moreover, a high-fat meal (about 910 Kcal, 57% of which from fat) has no significant effect on the rate and extent of absorption of tadalafil but decreases the rate of absorption for sildenafil and vardenafil, possibly affecting the onset of effectiveness [71, 72].

Recently avanafil, a new PDE5-I, was approved for marketing in Europe; this drug has shown to have advantageous properties, that is, a fast effect, about 35 min following its administration, and low side effects related to the combined treatment with nitrates and potential opportunities in elderly patients too (such as in patients who underwent a radical prostatectomy or affected with hyperglycemia or heart diseases). Avanafil has higher selectivity (120-fold) against PDE6 than sildenafil (16-fold) and vardenafil (21-fold) and high selectivity (>10,000-fold) against PDE1 compared with sildenafil (380-fold) and vardenafil (1000-fold); it does not inhibit PDE11 [73].

These drugs sometimes are not efficacious with the first dose, but results are generally improved with repeated dosing. On-demand treatment regimens have shown efficacy rates of 60–70% [74]. Furthermore, as above mentioned, 30–50% out of the patients that initially do not respond to PDE5-I may be converted to responders by counseling the patient and his partner [15]. Usual starting dose is 50 mg for sildenafil and 10 mg for vardenafil and tadalafil; doses may be increased up to 100 mg for sildenafil and 20 mg for vardenafil and tadalafil. Avanafil is available in 50, 100, or 200 mg tablets [73].

The year 2013 marked the 15th anniversary of the introduction of the first commercially available highly selective PDE5-I for ED; it also represents the year in which sildenafil's patent will start to expire throughout the world [2]. Sildenafil orodispersible tablets (50 mg) will be available in the next days.

Some patients who do not benefit from PDE5-I on-demand treatment may benefit from a daily low-dose administration [74–76]. This can be especially applied to elderly patients, where chronic administration can also work for other conditions frequently reported such as low urinary tract symptoms (LUTS). On the other hand, chronic administration of PDE5-I may improve erectile and endothelial responsiveness of men previously nonresponsive to on-demand regimens [11]. At this purpose, sildenafil may be used in a daily dose of 25 mg [77, 78], tadalafil is available in a daily dose of 2.5 and 5 mg, and vardenafil is available in a daily dose of 2.5 mg in some countries [15]. In a study performed for analyzing the efficacy and safety of sildenafil citrate in the geriatric population, these PDE5-I were found to be an effective agent in elderly men but with a lower efficacy rate especially in men aged >80 years old [79]. In this study 167 patients, mean age 72 ± 9 years old, were divided into three groups: 60–69, 70–79, and ≥80 years. Overall 54% of men responded to sildenafil, with a mean increase in International Index of Erectile Function (IIEF) domain score of 5.7. The incidence of adverse events (AEs) was similar to that in the general population taking sildenafil; importantly, no difference in AEs was found in the three age groups [79]. Furthermore, in those men affected with ED and late-onset hypogonadism, supplementation with testosterone may enhance the efficacy of PDE5-I [15]. This occurs for two main reasons: first, testosterone keeps the erectile tissue and the supplying nerves in a healthy condition and second it is widely known that testosterone increases the efficacy of PDE5-I by increasing the bioavailable NO in the cavernous smooth muscle [80, 81]. Furthermore, testosterone has been shown to be one of the main modulators of the expression of penile phosphodiesterase type 5 isoenzyme [11].

The main differences in the PDE5-I are in duration of action; tadalafil is able to potentiate sexual spontaneity by its longer half-life. In fact, its half-life is 17–21 h, whereas sildenafil and vardenafil half-lives are 4.6 h and 5 h, respectively. Avanafil presents a mean half-life of 5–10 hours [73].

Recently an orodispersible tablet (ODT) formulation of vardenafil has been developed, which dissolves in the subject's mouth (supralingual formulation) [82]. Vardenafil ODT is very advantageous because it has a 1.21-to-1.44-fold higher bioavailability than the film-coated tablet formulation (as measured by the area under the plasma concentration versus time curve (AUC)), and maximum concentrations in plasma after a single dose (Cmax) are comparable between the two formulations [82]. Therefore, the pharmacokinetics of ODT vardenafil is not equivalent to that of the film-coated tablets, because the ODT formulation provides greater vardenafil systemic exposure [83]. The primary objective of POTENT I (pivotal phase III trial to investigate the efficacy and safety of an orodispersible tablet vardenafil versus placebo in the treatment of men with erectile dysfunction: a fixed-dose, double-blind, randomized, multicenter trial) was to compare the efficacy and safety of on-demand 10 mg vardenafil ODT with placebo after 12 weeks of treatment or last observation carried forward (LOCF) in a general population of men with ED [82]. Importantly, the POTENT I study included a very large number of subjects aged ≥65 years (54.8%). 40 centers in Belgium, France, Germany, the Netherlands, Spain, and South Africa were involved in the study. This study demonstrated that 10 mg vardenafil ODT, taken on demand, improved erectile function and was well tolerated in a broad population of men with ED, irrespective of age. Vardenafil ODT definitely offers a more convenient therapeutic option for the treatment of ED, compared with the film-coated tablet formulation.

The POTENT II randomized trial was a double-blind, multicentre, randomized, parallel-group, placebo-controlled study conducted at 35 centres in Australia, Canada, Mexico, and USA. Subjects with ED for at least 6 months were randomized to receive 12 weeks of on-demand treatment with either 10 mg vardenafil ODT or placebo. Importantly, approximately half of the subjects were aged ≥65 years [84]. Of the 473 men enrolled in the study (51.4% aged ≥65 years), 331 were included in the intent-to-treat population (vardenafil ODT, *n* = 169; placebo, *n* = 162). Vardenafil ODT therapy was statistically significantly superior to placebo for all primary and secondary efficacy variables (*P* < 0.0001) [84]. Primary variables were International Index of Erectile Function (IIEF-EF) and Sexual Encounter Profile questions 2 (SEP2) and 3 (SEP3). Treatment with 10 mg vardenafil ODT, taken on demand, significantly improved erectile function and was effective and well tolerated. What these studies have added is that, compared with existing film-coated formulations, a more convenient method of taking vardenafil, in particular without the need for water or other liquids, may be absolutely preferable [83].

PDE5-I are also used in ED following nerve-sparing radical prostatectomy and in lower urinary tract symptoms (LUTS).

In a recent study acceptance and discontinuation data were analyzed in 100 consecutive, age-comparable, and preoperatively self-reported potent patients who underwent bilateral nerve-sparing radical prostatectomy (BNSRP) and at the hospital discharge received a PDE5-I treatment [85]. Medical and sexual history was collected on hospital admission and the IIEF was administered every 6 months postoperatively up to the 18-month follow-up. 49 patients freely decided not to start any treatment; 36 patients opted for an as-needed PDE5-I treatment, whereas 15 patients decided to use a daily PDE5-I. At the end of the study roughly 73% of patients who started therapy eventually discontinued it and a treatment effect below expectations was the main reason for stopping it [85].

The Multinational Survey of the Aging Male (MSAM-7) showed that roughly 50% out of the patients affected with moderate-to-severe LUTS present a concurrent ED, especially in those aged over 70 years old [86]. For those high numbers of patients PDE5-I might be a remarkable tool for treating both diseases; among the available drugs, tadalafil seems to be preferred for its well-known long half-life. The mechanism of action of these drugs in LUTS is not quite clear; however, PDE5 is found in prostate, bladder, urethra, and their respective blood vessels. A crucial role is also played by NO, which is able to mediate urinary function through a number of pathways. However, PDE5-I can potentially improve LUTS by increasing cGMP, which is the final mediator in the NO pathway [87, 88].

Furthermore, the safety profile of the currently available PDE5-I is excellent in the elderly too. No increased rate in myocardial infarction was found in elderly patients receiving these agents compared to expected rate in age-matched populations [70]. Of course, as well as in younger patients, PDE5-I are contraindicated in patients with unstable angina pectoris, recent myocardial infarction, some arrhythmias, and poorly controlled hypertension [15]. Headache, facial and ocular hyperaemia, nasal congestion, and back pain are commonly reported following PDE5-I treatment. Cross reactivity with PDE6 in the retina causes visual blurring [15]. Flushing and dyspepsia are sometimes reported in elderly patients treated with sildenafil and tadalafil [88, 89]. Furthermore, vision loss due to nonarteritic ischemic optic neuropathy was associated with the use of PDE5-I [2]; people of 50 years old or older, affected with hypertension and/or heart disease, diabetes, hypercholesterolemia, and smokers are more usually involved.

Drug-disease interactions and pharmacodynamic interactions are potentially dangerous; therefore, patients who are treated with nitrates or nitrate donors should not take PDE5-I, except for avanafil, which seems to be safer even when used with these drugs concurrently [73]. Past use of nitrates, usually >2 weeks, is not a contraindication but a period superior to 24 hours for short acting PDE5-I (sildenafil and vardenafil) and superior to 48 hours for long-acting tadalafil is strictly recommended before taking nitrates [40]. Short acting PDE5-I such as sildenafil, vardenafil, and avanafil should also be preferred in patients affected with cardiovascular disease [43]. Concurrent treatment of PDE5-I and *α*-blockers might result in postural hypotension and needs to be carefully assessed. Precaution is suggested in the use of labetalol and carvedilol, which present mixed *α*- and *β*-blocker activity [66].

However, when doses of 100, 150, and 200 mg of sildenafil (doses in excess of the recommended range) were administered in a group of healthy but young volunteers, the mean maximum decrease in standing systolic blood pressure was −10/−7 mmHg, with the maximum change occurring 3 hours after dosing [90]. None of the PDE5-Is are dangerously associated with QTc prolongation, although vardenafil has a warning for patients at risk for QTc prolongation [90]. However, it has been shown that a large range of vardenafil doses/concentrations (up to 80 mg/day) may produce a small QTc prolongation that is not associated with absolute QT prolongation [91]. This prolongation is similar to that observed with sildenafil and is unlikely to be clinically relevant [92]. On the contrary, vardenafil is not recommended in patients taking type 1A antiarrhythmics (such as quinidine or procainamide) or type 3 antiarrhythmics (amiodarone or sotalol) [91, 93].

Elderly people often take several drugs; when taking PDE5-I, one should be careful to potential pharmacokinetic interactions via cytochromes (CYP) [94]. Sildenafil is mainly metabolized through CYP3A4 and secondarily via CYP2C9. This means that drugs inhibiting or inducing these enzymes may lead to, respectively, an increase or a reduction in plasma concentrations of sildenafil (Table 1) [94]. Concurrent administration of sildenafil and warfarin may potentially lead to an increased risk of bleeding. In fact,* in vitro* studies have also shown that sildenafil is a weak inhibitor of CYPs 1A2, 2C19, 2D6, 2E1, and 3A4. Tadalafil and vardenafil are mainly metabolized by CYP3A4; all the inhibitors of CYP3A4 (ketoconazole, fluconazole, ritonavir, indinavir, amiodarone, erythromycin, fluoxetine, fluvoxamine, omeprazole, and grapefruit juice) increase the area under the concentration curve (AUC) of tadalafil and vardenafil (Table 1). Drugs prolonging QTc interval should be cautiously administered together with vardenafil for the potential risk of further increase in QTc (Table 1) [91, 93].

#### 6.4.2. Other Pharmacological Treatments for ED

In the late years a number of herbal and nutritional supplements have been used in the treatment of ED, even if they lack strong evidence, for example, yohimbine, icariin, and ginseng [15]. Yohimbine is a peripherally and central *α*_2_-blocking agent derived from the bark of an evergreen tree; it is also a mild monoamine oxidase inhibitor (MAOI). It blocks the pre- and postsynaptic *α*_2_ receptors, but in particular the blockade of *α*_2_ receptors facilitates the release of several neurotransmitters in both the central and peripheral nervous system and the corpus cavernosum, such as nitric oxide and norepinephrine [15, 95]. It can be used at dosages of 15 mg/day (5 mg three times a day) or 15 mg 1-2 hours before sexual activity together with 6 g arginine glutamate (50% arginine, 50% glutamic acid) [96]. It is not used in elderly people because of the possible side effects, such as hypertension, tachycardia, anxiety, insomnia, hallucinations, and dizziness.

Icariin is a flavonol glycoside derived from horny goat weed or Herba Epimedii, used for centuries in China for enhancing sexual performances; it can have an inhibitory effect of both PDE4 and PDE5, thus enhancing the production of bioactive nitric oxide and mimicking the effects of testosterone [97–99].

Ginseng is another widely known aphrodisiac which can increase erectile function, even if data are preliminary and need further trials [15].

The vacuum constriction device is a manually operated device that creates negative pressure around the penis, thus resulting in passive engorgement of the sinusoidal spaces and erection; the maintenance of the erection is facilitated by a rubber cuff applied around the penile base [15]. It is used in men who do not wish medical treatment or penile implantation surgery or do not respond to medical treatment. It is effective up to 90% of patients, even if the turgidity occurs distal to the constriction band; the use of the constriction band is possible for 30 min in order to avoid skin necrosis [100] and it has to be used cautiously in patients taking anticoagulants or presenting with bleeding disorders [101].

The intracavernous and intraurethral administration of vasoactive substances is the recommended second-line treatment in patients that fails to respond to PDE5-I for ED; it is safe, effective (up to 80% out of the cases), and with a rapid onset of action [15]. The most common vasoactive substances are prostaglandin E1 (alprostadil or PGE1), papaverine, and phentolamine. PGE1 and papaverine increase the intracellular concentration of the second messenger cGMP and cyclic adenosine monophosphate, thus resulting in cavernous smooth muscle relaxation, whereas phentolamine is an *α*-adrenergic antagonist [15].

Alprostadil (PGE1) is also available as an intraurethral administrated pellet (medicated urethral suppository for erection (MUSE)). The medicine is a small pellet contained inside a thin tube that is inserted into the urethra. The pellet can be released by pressing a button on the applicator and an erection develops in about 10 minutes and lasts at least 30 minutes, but usually less than 60 minutes, depending on the dosage [15].

Side effects of MUSE are usually minor and may include pain in the penis or urethra, mild injury to the urethra, such as a small scrape that produces a drop of blood at the tip of the urethra, and priapism. If an erection lasts longer than 3 hours and it is not relieved, it may damage tissues inside the penis.

The MUSE system does not cause bruising or scar tissue, like injections may. Partners of men who have vision problems or who may have difficulty inserting the pellet can be taught how to use these products. The medicine may cause irritation for partner after ejaculating [15, 101, 102].

#### 6.4.3. Surgical Procedures

Surgical treatment is indicated in elderly patients in which conservative treatments for ED have failed or those declining pharmacotherapy; it usually consists of penile prosthesis. There are three types of penile prosthesis, but the three-piece inflatable prosthesis is the preferred device. It consists of two implantable rods, connected to a pump device placed in the scrotum and a reservoir which is placed in the preperitoneal space in the lower abdomen [15]. It has a 90–98% satisfaction rate [100]. Possible adverse events are infections, occurring in 1-2% of the patients, autoinflation, and erectile length loss, especially in patients with Peyronie's disease and after radical prostatectomy.  Table 2 summarizes the possible treatment options for ED in elderly patients.

## 7. Recreational Use of ED Medications in the Elderly 

Use of ED medication could result in men experiencing “ideal” erections that are both firmer and more durable. This even occurs in the elderly, especially in healthy young elderly, where keeping a normal sexual life may be a marker of healthy and successful aging.

Sometimes the use of ED medications as an exclusive therapy can reveal or reinforce other sexual problems, such as a lack of sexual desire and premature ejaculation. In most cases, recreational use concerns young people: in fact, in a previous study by Harte and Meston, 1,944 men, recruited from 497 undergraduate institutions across the USA, were asked about recreational PDE5-I and illicit drug use. Surprisingly, 4% of the study participants had recreationally used PDE5-I at some point in their lives and one-third of them reported current use. Most of them (44%) reported mixing PDE5-I with alcohol or illicit drugs, particularly while engaging in risky sexual behaviour. Illicit drugs taken concomitantly with PDE5-I included marijuana (61%), 3,4-methylenedioxy-N-methylamphetamine (MDMA; 42%), methamphetamines (36%), cocaine (30%), alkyl nitrites, and ketamine [103, 104]. On the other hand, approximately 6 million men in Europe might currently bypass the healthcare system to obtain PDE5-I [105]. In addition to the possible risks associated with the use of PDE5-I from uncontrolled sources, because most of these men have ED, they also miss the opportunity of important health information or medical follow-up.

However, use of recreational PDE5-I is independently associated with increased age, drug abuse, lifetime number of sexual partners, and lifetime number of “one night stands,” as well as with homosexual or bisexual orientation [106].

As above mentioned even elderly men can use ED medications only for improving sexual performances, especially PDE5-I; moreover, PDE5-I can at the same time improve LUTS. Young-old people, that is, people aged between 65 and 74 years old, can be mostly concerned in the recreational use of PDE5-I.

## 8. Future Perspectives

A man's ability to get and sustain an erection is often equated with virility and masculinity and is able to greatly affect men's self-esteem.

The availability of PDE5-I has significantly altered the way in which ED is treated. Furthermore, it has been shown the possible role of these drugs in synaptic function and in memory in an Alzheimer's disease mouse model of amyloid deposition [107]. In fact, sildenafil is able to enhance phosphorylation of cAMP response element binding protein (CREB), a molecule involved in memory, through elevation of cGMP levels [107].

Sildenafil can be also used in medical conditions different from ED, such as pulmonary arterial hypertension [108]. It has also been shown to be effective in treating severe Raynaud's phenomenon associated with systemic sclerosis and digital ulceration. Investigative studies have suggested that sildenafil has also promise in the treatment of respiratory disorders with ventilation/perfusion mismatch, congestive cardiac failure, hypertension, and even stroke [108].

Currently available oral pharmacotherapy has limited efficacy in older patients due to the lack of endogenous supply; some new compounds are currently in development, such as guanylate cyclase activators, Rho-kinase inhibitors, and maxi-K channel openers [15].

The use of stem cells for the treatment of ED represents an exciting new field, which still requires extensive basic research and human trials in diverse ED patient populations in order to define its role in the treatment of ED [109]. Preclinical studies have shown that these cells may reverse pathophysiological changes leading to ED, for example, following cavernous nerve injury and in Peyronie's disease, diabetes, aging, and hyperlipidemia. Overall, these studies have shown beneficial effects, while evidence on the mechanisms of action of stem cell therapy still varies between studies [109].

## 9. Concluding Remarks

In conclusion, increasing comorbidities and pathological changes in the erectile tissue and the supplying vessels result in a high prevalence of ED in the geriatric population.

ED is a multifactorial condition and, as such, prevention and treatment demand a multidisciplinary approach. Of course, it is always important for the physician to avoid the use of any medication to improve sexual activity if the underlying cardiac condition of the patient does not permit activity appropriate for sexual activity. At the same time, before starting a treatment for ED, physicians should always review all drugs potentially worsening erectile function; an accurate counseling should always be carried out. Various nonpharmacological, pharmacological, and surgical options are safe and effective in the elderly. PDE5-I are safe and usually effective drugs also in the elderly. Data acquired during a routine diagnostic workup for ED should be taken into account when choosing the best PDE5-I for the individual patient. An individualized treatment plan is recommended and going beyond “experience-based” subjective opinion and unfounded ideas and prejudice regarding currently available drugs may be appropriate [40]. Vardenafil orodispersible preparation is reported to have the fastest onset of action, while tadalafil is reported to have the longer duration of action. PDE5-I can also be used for indications other than ED (sildenafil in pulmonary hypertension and tadalafil in LUTS) and chronic administration seems to work better in elderly patients. New hopes might derive from the use of avanafil. Novel compounds are currently in development and are expected to have better efficacy in nonresponders. Future research will be soon available for improving the sexual health of older men.

## Figures and Tables

**Table 1:** Some examples on potential interactions among PDE5I and other drugs via CYP450.

Cytochrome	Substrates	Inhibitors	Inductors
CYP1A2	*PDE5I: *sildenafil Antidepressants: tricyclics, fluvoxamine, mirtazapine Antipsychotics: haloperidol, olanzapine, clozapine Methylxantines: caffeine, teophylline Other drugs: R-warfarin, tacrine, paracetamol	Ciprofloxacin Fluvoxamine	Barbiturates Carbamazepine Phenytoin Rifampicin Tobacco

CYP3A4	*PDE5I: *sildenafil, tadalafil, vardenafil, Antidepressants: tricyclicics, venlafaxine, citalopram, mirtazapine Benzodiazepines: diazepam, bromazepam Nonbenzodiazepine anxyolitics: buspirone Antipsychotics: haloperidol, clozapine, risperidone, ziprasidone, sertindole, quetiapine, aripiprazole Anticonvulsants: carbamazepine, felbamate, tiagabine Calcium-antagonistis: nifedipine, diltiazem, verapamil Other drugs: amiodarone, quinidine, terfenadine, astemizole, macrolides (erythromicin, clarithromycin), tamoxifen, cyclosporine,	Antiarrhytmics: quinidine, amiodarone Antifungal drugs (fluconazole, itraconazole, ketoconazole) Antidepressants: fluvoxamine, fluoxetine, nefazodone Antihistamines: loratadine Antiviral drugs: indinavir, ritonavir Grapefruit juice (at least 250 mL) Macrolides: clarithromycin Erythromicin Calcium channel blockers: diltiazem, verapamil Proton pump inhibitors: omeprazole, testosterone	Barbiturates Carbamazepine Felbamate* Hypericum Oxcarbazepine* Phenytoin Rifampicin Topiramate* *Weak enzymatic inductor

*refers to the activity of felbamate, oxcarbazepine and topiramate as weak enzymatic inductors.

**Table 2:** Summarizing the possible treatment options for ED in elderly patients.

Drug	Dose	*t*_1/2_ (h)	Frequency	Advantages	Side effects
Sildenafil Tadalafil Vardenafil Avanafil	25, 50*, 100 mg 2.5, 5, 10, 20 mg 2.5, 5, 10*, 20 mg 50, 100, 200 mg	4.6 17–21 4-5 5–10	On demand or daily	Safe; available on demand as well as continuous low dose (vardenafil 2.5 mg, tadalafil 2.5 mg)	Headache, myalgia, back pain, blurred vision, facial flushing, nasal congestion, dizziness

Yohimbine	5–15 mg	0.25–2.5	Three times daily	Natural product	Hypertension, tachycardia, anxiety

Vacuum constriction device	/	/	On demand	Effective in 90% of patients; not expensive	Skin necrosis, pai006E, cold penis, unnatural erection

Papaverine PGE1 Phentolamine	30–110 mg 5–40 *μ*g 1.25–2 mg	1.5–2.5 0.30 0.19	On demand	Broad efficacy, safety, and effecacy in neurogenic ED	Priapism, pain, penile fibrosis, injection training requested

MUSE	125, 250, 500, 1,000 *μ*g	0.30	On demand	No injections needed	Hypotension, pain, urethral burning, syncope, vaginal irritation in the partner

Penile prosthesis	/	/	On demand	High satisfation rates	Irreversible, infection, erectile lenght loss, autoinflation

*t*_1/2_: plasma half-life; h: hours; *also available in orodispersable formulation (supralingual).
